# Signature miRNAs Involved in the Innate Immunity of Invertebrates

**DOI:** 10.1371/journal.pone.0039015

**Published:** 2012-06-19

**Authors:** Geng Yang, Lu Yang, Zhe Zhao, Jiajia Wang, Xiaobo Zhang

**Affiliations:** Key Laboratory of Conservation Biology for Endangered Wildlife of Ministry of Education, Key Laboratory of Animal Virology of Ministry of Agriculture and College of Life Sciences, Zhejiang University, Hangzhou, The People’s Republic of China; Instituto Butantan, Brazil

## Abstract

The innate immune system, including the cell-based immunity (mainly apoptosis and phagocytosis) and the humoral immunity (such as pro-phenoloxidase system), is the first defense line of animals against the infection of pathogens in a non-specific manner, which is fine regulated through the gene expression regulations. The microRNAs (miRNAs) are recognized as important regulators of gene expression. To date, however, a comprehensive view about the regulation of innate immunity by miRNAs is not available. To address this issue, the signature miRNAs involved in the innate immunity were characterized in this study. The phagocytosis, apoptosis and phenoloxidase (PO), a key enzyme in the pro-phenoloxidase system, of invertebrate shrimp were activated or inhibited, followed by the small RNA sequencing. The results showed that a total of 24 miRNAs took great effects on phagocytosis, apoptosis or the pro-phenoloxidase system, which were further confirmed by Northern blots. Among the 24 innate immunity-associated miRNAs, 21 miRNAs were conserved in animals, suggesting that these miRNAs might share the similar or the same functions in different species of animals. Based on degradome sequencing and prediction of target genes, it was found that the miRNAs might mediate the regulations of phagocytosis, apoptosis or pro-phenoloxidase system by targeting different genes. Therefore our study presented the first comprehensive view of the miRNAs associated with innate immunity, which would facilitate to reveal the molecular events in the regulation of innate immunity.

## Introduction

It is well known that host immune responses to pathogens depend on the immune system. Highly developed animals have developed a complex system of checks and balances for immune regulation in order to maintain self tolerance while allowing immune responses to foreign pathogens. Innate immunity and acquired immunity are the two major parts of host defense approaches [1]. The acquired immunity exists only in vertebrates, which has been well elucidated [2]. As the first immune defense line of animals, the innate immunity functions in vertebrates and invertebrates by mediating recognition of non-self and activating, the corresponding immune responses [3]. The innate immunity, controlled by genetic factors with relative stability, becomes a very effective defense system of animals against the invasion of pathogens. Comparing with the acquired immunity, however, the innate immunity and its regulation are not intensively investigated [4]. As well known, the microRNAs (miRNAs) play very important roles in gene expression regulations. Recently, it is evident that the miRNAs are involved in the immune responses.

The miRNAs are endogenous non-coding RNAs with approximately 22 nucleotides (nt) in length. Their biogenesis starts with transcription of miRNA genes, which are further processed by Drosha/DGCR8 and Dicer [5], [6], [7]. The mature miRNA strand is incorporated in the RNA-induced silencing complex (RISC), serving as a leading RNA to control the expression of cognate mRNA for degradation or translation repression. Given their roles in regulating gene expression, it is not surprising that miRNAs have been exhibited to be involved in a wide variety of biological processes [8]. Dicer is a key enzyme in the generation of miRNAs. It is reported that the deletion of Dicer at the early B cell stage leads to the inhibition of the pro- to pre-B cell transition which coincides with a significant up-regulation of the pro-apoptotic protein Bim [9]. Granulocytes, monocytes and natural killer (NK) cells provide important first lines of defense against pathogen infection. Emerging data have identified contributions of miRNAs to the development and function of these innate immune cells. The miR-223 and miR-424 can promote monocyte and neutrophil differentiation by repressing the expression of nuclear factor I/A (NFI-A) [10], [11], while the miR-34 and miR-21 repress the mRNAs encoding WNT1 and Jagged 1 (JAG1) to promote DC differentiation [9], [12]. It is evident that the growth factor independent 1 (GFI1) represses the expressions of miR-196b and miR-21 during granulocyte development [9]. As one of the greatest important and muli-roles during the innate immune response, the miR-155 is well documented. It is found that the miR-155 can enhance the production of TNF-α, suggesting the positive role of miR-155 to regulate the release of inflammatory mediators [13], [14], [15], [16], [17]. In the miR-155 knock-out mice, the miR-155 is verified to be required for the normal immune function. The miR-155 can also repress the expressions of suppressors of cytokine signaling 1 (SoCS1) and SHIP1, which are the negative regulators of the Toll-like receptor pathway. The data about the immune regulation by miRNAs are accumulated. To date, however, we have not yet achieved a comprehensive view of the regulation of innate immunity by miRNAs.

To address this issue, the miRNAs of shrimp and their targets were characterized in this study by miRNA sequencing and degradome sequencing. Degradome sequencing, also referred to as parallel analysis of RNA ends (PARE), allows the globe-wide analysis of miRNAs mediating cleavage events in organisms. This method becomes an efficient approach used for the analysis of miRNA targets. Shrimp is one of the most important species in marine aquaculture. The non-specific innate immunity of shrimp, including their humoral defenses (mainly pro-phenoloxidase system) and their cellular defenses (phagocytosis and apoptosis), is the sole mechanism for them to defend themselves against invading pathogens [18], [19], [20], [21]. In this investigation, the miRNAs and their pathways involved in the innate immunity of shrimp (phagocytosis, apoptosis and pro-phenoloxidase system) were characterized. Our study presented the first comprehensive view of the regulation of innate immunity by miRNAs.

## Materials and Methods

### Shrimp Culture

*Marsupenaeus japonicus* shrimp, about 5 g and 5–7 cm each, were maintained in different groups of 15 individuals each in 80 L aquariums filled with air-pumped seawater at 20°C.

### The Inhibition or Activation of Apoptosis of Shrimp Hemocytes

The shrimp were injected with apoptosis inhibitor z-vad-fmk or apoptosis inducer cycloheximide at different concentrations to block or activate the apoptosis of shrimp hemocytes, respectively. As control, the shrimp were injected with DMSO only. At various time after injection, the shrimp hemolymph was withdrawn with equal volume of heparin sodium (40 mg/ml) and used for determination of caspase3/7 activity according to the manufacturer’s instruction (Promega, Madison, WI, USA). Briefly the shrimp hemolymph was plated in 96-well plate in triplicate. After incubation with equal volume of caspase substrate for 2 h at room temperature, the samples were read on a SpectraMax Gemini M5 Microplate luminometer (Molecular Devices, Sunnyvale, CA, USA).

To confirm the inhibition or activation of apoptosis by inhibitor or inducer, the terminal deoxynucleotidyl transferase dUTP nick end labeling (TUNEL) assays were conducted according to the manufacturer’s manual (Promega, USA). Briefly, shrimp hemolymph was mounted onto a poly-L-lysine-coated glass slide (Sigma, USA), followed by incubation for 10 min at 4°C. The hemocytes were fixed with 4% methanol-free paraformaldehyde for 10 min at 4°C after removing the supernatant. Subsequently the hemocytes were rinsed twice with phosphate-buffered saline (PBS) and permeablized with 0.2% Triton X-100 solution in PBS for 5 min. After rinsing slides with PBS twice at room temperature, cells were covered with 100 µl of Equilibration Buffer at room temperature for 10 minutes. The cells were incubated at 37°C for 60 min with rTdT mix containing green fluorescein-12-dUTP. Then the cells were counterstained with propidium iodide (PI). The reactions were terminated by immersing the slides in 2×SSC for 15 min. After washes by PBS twice, the slide was air-dried and mounted with anti-fade solution (Invitrogen, USA) to assess fluorescence by microscopy.

### The Inhibition of Phagocytosis of Shrimp Hemocytes

The shrimp were injected with phagocytosis inhibitor (cytochalasin B) or DMSO as control at different concentrations. At various time after the injection of inhibitor, the shrimp hemocytes were collected and subjected to phagocytic activity detection. The phagocytic activity was assayed as described previously. Briefly, the shrimp hemolymph was incubated with FITC-labeled WSSV (white spot syndrome virus of shrimp) virions at 28°C for 30 min. Subsequently the mixture was smeared onto a poly-L-lysine-coated glass slide and allowed to fix for 15 min at room temperature. The hemocytes were stained with propidium iodide (PI) (Sigma, USA) at the final concentration of 20 mg/ml for 1 min. Then, the non-adherent cells were removed with PBS, followed by the addition of Trypan blue to quench any free floating and adherent WSSV. After incubation for 20 min, the cells were washed once with PBS and one drop of anti-fade solution (Invitrogen, USA) was added before mounting to examine phagocytic activity with a flow cytometer or a fluorescence microscope. A total of 500 hemocytes were examined and phagocytic activity was expressed as the percentage of cells showing fluorescence.

### The Inhibition or Activation of Phenoloxidase of Shrimp Hemocytes

To inhibit or activate the phenoloxidase activity, the shrimp were injected with phenoloxidase inhibitor (allylthiourea) or activator (N-Tosyl-l-phenylalanine chloromethyl ketone, TPCK) at different concentrations. As control, the shrimp were injected with DMSO only. At various time after the injection of inhibitor or activator, the shrimp hemolymph was collected and subjected to phenoloxidase activity detection. The shrimp phenoloxidase activity was spectrophotometrically determined as described previously with minor modifications. Briefly, the shrimp hemolymph was withdrawn with equal volume of heparin sodium (40 mg/ml) and processed by low-speed centrifugation at 500 g for 3 min to remove cells and tissue debris. Subsequently the cell-free hemolymph was transferred to a tube containing L-Dopa buffer (3 mM L-Dopa in 10 mM Tris–HCl, pH7.2). After incubation at 37°C for 20 min, the amount of dopachrome produced in the reaction was determined at OD_490_. The optical density of the shrimp phenoloxidase activity was expressed as dopachrome formation in 10 µl of cell-free hemolymph. The L-Dopa buffer only was used as control.

### Extraction of Total RNAs and Sequencing and Analysis of Small RNAs

Total RNAs were extracted from the hemocytes of shrimp treated with the inhibitors or activators of apoptosis, phagocytosis or phenoloxidase using mirVana miRNA Isolation Kit (Ambion, Austin, USA) according to the manufacturer’s protocol. Subsequently 200 µg of total RNAs was separated onto a denaturing 15% polyacrylamide gel. The small RNAs ranging from 16 to 30 nt were excised and dephosphorylated by alkaline phosphatase. After recovery by ethanol precipitation, the purified small RNAs were ligated sequentially to RNA adapters (5′- ACAGGUUCAGAGUUCUACAGUCCGACGAUC-3′ and 5′-UCGUAUGCCGUC UUCUGCUUG-3′). Reverse transcription and PCR amplification were preformed after ligation. The resulting products were sequenced on the Genome Analyzer GA-I (Illumina, San Diego, USA) according to the manufacturer’s instructions.

The small RNA sequences were processed using Illumina’s Genome Analyzer Pipeline software (Illumina) and then subjected to a series of data filtration steps using the ACGT101-miR program developed by LC Sciences (USA). After the removal of the raw sequences and those matched with the sequences of vector, mRNA and Rfam (rRNA, tRNA, snRNA, snoRNA and repeat sequence), all the trimmed sequences between 16 and 26 bp in length were searched against the known mature and precursor miRNAs in miRbase 15.0 to check whether these miRNAs were homologous with those of other species. To reveal the shrimp miRNAs with no homologue in animals, the miRNAs were analyzed by BLASTN search against the shrimp expressed sequence tag (EST) database from The National Center for Biotechnology Information (NCBI), allowing one mismatch between two sequences. After removal of the protein-coding EST sequences, the remaining non-coding candidate shrimp ESTs with perfect matches with miRNA sequences were used for hairpin structure prediction from the adjacent 60 nt sequences in either direction complying with criteria of the statistics of mammalian pre-miRNAs in miRBbase 15.0 using UNAfold software. The hairpin structures were one of the key features to distinguish miRNAs from other endogenous small non-coding RNAs.

### Northern Blot Analysis

Total RNAs were extracted from shrimp hemolymph using a commercial kit (Ambion, USA) according to the manufacturer’s instructions. After separation on 15% polyacrylamide gel containing 7 M urea gel, the RNAs were transferred to a nylon membrane (Amersham Biosciences, UK). The miRNAs and U6 were detected with a digoxigenin (DIG)-labeled-DNA probe complementary to a specific miRNA sequence. The DIG labeling and detection were performed following the manual of DIG High Prime DNA Labeling and Detection Starter Kit II (Roche, Grenzacherstrasse, Basel, Switzerland).

### Degradome Sequencing and Analysis

The total RNAs were extracted from shrimp hemocytes using a commercial kit (Ambion, USA). Subsequently the polyadenylated RNAs were isolated from the total RNAs using oligo(dT) dynabeads (Ambion, USA), followed by ligation of the polyadenylated RNAs with an RNA oligonucleotide adaptor (5′-GUUCAGAGUUCU ACAGUCCGAC-3′) which could only bind with 5′-phosphorylated-dependent RNA strands (Ambion). The ligated products were purified using oligo(dT) dynabeads and used as templates in a reverse transcription reaction with the primer (5′-CGAGCAC AGAATTAATACGACT-3′). The resulting products were amplified by PCR using primers (5′-GTTCAGAGTTCTACAGTCCGAC-3′) and (5′-CGAGCACAGAATTA ATACGAC-3′) under conditions of 7 cycles of 94°C for 30 s, 60°C for 20 s and 72°C for 3 min. The PCR products were gel purified, cleaved with Mme I (New England Biolabs) and dephosphorylated. Then the samples were purified and ligated using T4 DNA ligase (Ambion) with double-stranded DNA adaptors (5′-P-TCGTATGCCGTC TTCTGCTTG-3′ and 3′-NNAGCATACGGCAGAAGACGAAC-5′). After separation on 12% polyacrylamide gel, the 92 nt bands were excised and purified, followed by PCR amplification using the conditions of 18 cycles of 94°C for 20 s, 60°C for 20 s and 72°C for 20 s. The products were separated on polyacrylamide gel and purified. Then they were subjected to 35-base read high-throughput sequencing using the Illumina GA platform.

Raw reads were processed to remove adaptor sequences and the reads with sizes of 20 or 21 nt were retained. Then the Reads that did not correspond to structural RNAs (rRNA, tRNA, snRNA and snoRNA) were mapped to the shrimp expressed sequence tag (EST) database from The National Center for Biotechnology Information (NCBI). The abundance of reads that matched more than one transcript was normalized and the polyN sequences in the reads were omitted for further analysis. The identified degradation fragments of mRNAs were annotated into several categories based on priority and the software pairfinder (Beijing Genome Institute, China) was used to pair the miRNA-mRNA degradations.

### The Prediction of Target Genes of miRNAs

To predict the genes targeted by miRNAs, two computational target prediction algorithms (TargetScan 5.1 and miRanda) were used. The databases were the shrimp EST sequences and the *Drosophila megalogaster* genome sequence (NCBI). The TargetScan tool was used to search for miRNA seed matches (nucleotides 2∼8 from the 5′-end of miRNA) in the 3′ UTR sequences. The miRanda tool was able to match the entire miRNA sequences. The miRanda parameters were set as free energy <−20 kcal/mol and score >50. Finally, the data predicted by both algorithms were combined and the overlaps were calculated.

### Statistical Analysis

The numerical data were analyzed by one-way analysis of variance to calculate the mean and standard deviation of triplicate assays.

## Results

### The Dosages and Effective Time for Inhibitors or Activators of Apoptosis, Phagocytosis or Phenoloxidase

As reported, the phagocytosis, apoptosis and pro-phenoloxidase system were the major players in the innate immunity. To achieve a comprehensive view of the roles of miRNAs involved in innate immunity, the phagocytosis, apoptosis and phenoloxidase (PO), a key enzyme in the pro-phenoloxidase system, of invertebrate shrimp were activated or inhibited, respectively, followed by the small RNA sequencing.

**Figure 1:**
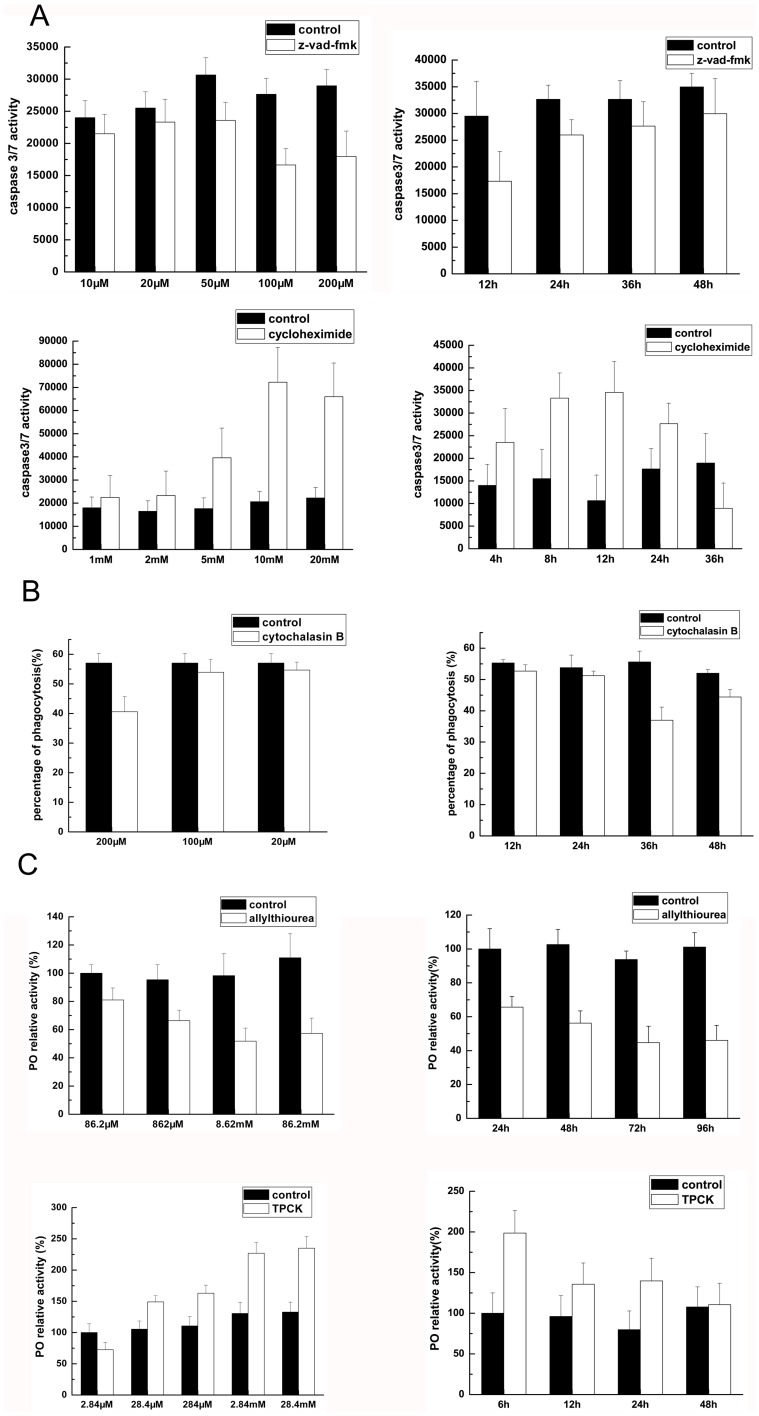
The dosages and effective time for inhibitors or activators of apoptosis, phagocytosis or phenoloxidase. (**A**) The dosage and effective time of apoptosis inhibitor z-vad-fmk and inducer cycloheximide. The shrimp were injected with the inhibitor or inducer at various concentrations, respectively. At different time after injection, the caspase 3/7 activity of shrimp hemocytes was detected. The hours indicated the time after the injection. Control, the shrimp without the apoptosis inhibitor or inducer. (**B**) The dosage and effective time of phagocytosis inhibitor cytochalasin. The shrimp were injected with the inhibitor at different concentrations, respectively. At different time after injection, the hemocytic phagocytosis activity was evaluated with the FITC-labeled WSSV virions. The hours indicated the time after the injection. Control, the shrimp without the phagocytosis inhibitor. (**C**) The dosage and effective time of phenoloxidase inhibitor and activator. The shrimp were injected with the inhibitor or activator at different concentrations, respectively. At various time after injection, the phenoloxidase activity of shrimp hemocytes was evaluated. The hours indicated the time after the injection. Control, the shrimp without the phenoloxidase inhibitor or activator.

To inhibit the apoptotic activity, the shrimp were injected with the apoptosis inhibitor z-vad-fmk at different concentrations. The detections of caspase3/7 activity showed that the inhibitor at 100 µM exhibited the highest inhibitory effect (Fig 1A). After the injection of z-vad-fmk at 100 µM, it was found the inhibitor presented the best inhibitory ability at 12 h (Fig 1A). In order to evaluate the suitable dosage of apoptosis inducers, the cycloheximide at various concentrations was injected into shrimp. The results showed that the inducer obtained the maximum activation activity at 10 mM (Fig 1A). It was revealed that the inducer demonstrated the best apoptosis- inducing effect at 12 h after the injection of cycloheximide (Fig 1A). The above apoptotic activities were further confirmed by TUNEL assays. At the best concentration and effective time of apoptosis inhibitor and inducer, the small RNAs of shrimp hemocytes were sequenced. Meanwhile the shrimp without apoptosis inhibitor and inducer was used as control in the small RNA sequencing.

In an attempt to investigate the miRNAs associated with phagocytosis, the shrimp hemocytic phagocytosis against WSSV was inhibited by the injection of cytochalasin B. After injections of cytochalasin B at different concentrations, the phagocytic activities of shrimp hemocytes were evaluated using the FITC-labeled WSSV virions. It was found that the best dosage for the injection of phagocytosis inhibitor was 200 µM (Fig 1B). The time course results showed that the phagocytosis inhibitor presented the highest inhibitory effect at 36 h after the injection of cytochalasin B (Fig 1B). Therefore the shrimp injected with cytochalasin B at 200 µM were collected at 36 h after the injection of phagocytosis inhibitor and subjected to the small RNA sequencing. The shrimp without phagocytosis inhibitor was included in the sequencing.

To determine the optimal concentration and time of the inhibitory effect of allylthiourea on phenoloxidase, the shrimp were injected with the inhibitor at different concentrations. Twenty four hours later, the phenoloxidase activity of hemocytes was measured. The result showed that the inhibitory effect of allylthiourea was dose-dependent (Fig 1C). When the concentration of allylthiourea reached 8.62 mM, the inhibitory effect did not change, indicating that it was the best dosage. Based on the time course data, it was found that the inhibitor exhibited the best effect at 72 h after the injection of allylthiourea (Fig 1C). For the activation of phenoloxidase, N-Tosyl-l-phenylalanine chloromethyl ketone (TPCK) was injected into shrimp with different concentrations. The results showed that the phenoloxidase activity was significantly stimulated when the concentration of TPCK was 2.84 mM (Fig 1C). The time course data indicated that the activator exhibited the best activation activity at 6 h after the injection of TPCK (Fig 1C). At the best concentration and effective time of phenoloxidase inhibitor and activator, the small RNAs of shrimp hemocytes were sequenced. The shrimp without phenoloxidase inhibitor and activator was used as control in the small RNA sequencing.

### Sequence Analysis of miRNAs Associated with Innate Immune Responses

Based on the small RNA sequencing, the small RNA sequences of shrimp treated with inhibitors or activators of apoptosis, phagocytosis or phenoloxidase were analyzed. The treatment-free shrimp were included in the analysis as controls.

**Figure 2:**
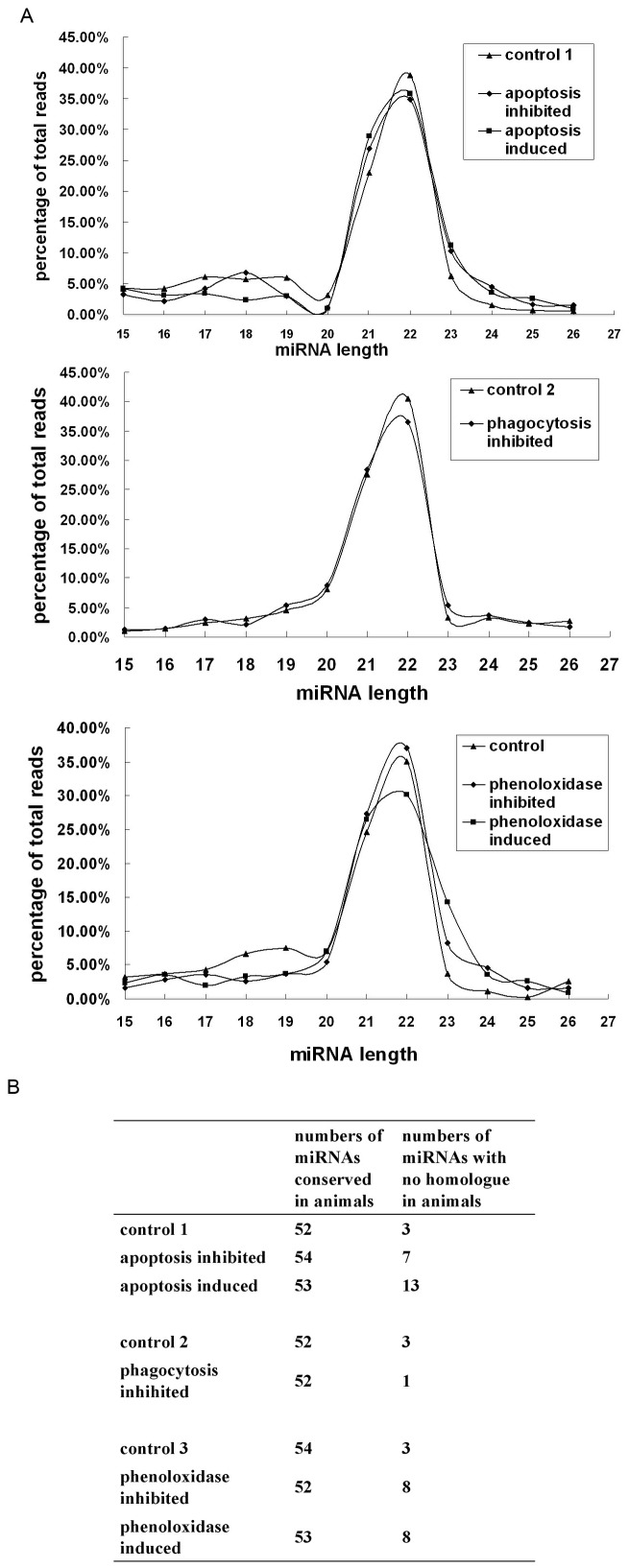
The shrimp miRNAs associated with innate immunity. (**A**) Length distribution of sequenced small RNAs associated with apoptosis (up), phagocytosis (middle) and phenoloxidase (down). The shrimp were treated with the inhibitors or activators of apoptosis, phagocytosis or phenoloxidase, followed by small RNA sequencing. Controls 1, 2 and 3, treatment-free shrimp. (**B**) Numbers of miRNAs conserved or without homologue in animals.

Totally the small RNA sequencing generated 9–12 million raw reads. About 83–91% raw reads were present at least twice and their lengths were ranged from 16 to 25 nucleotides. After removal of mRNA, rRNA, tRNA, snRNA and snoRNA, the high throughput sequencing generated a total of 2 million sequences. The data analyses showed a low proportion of long RNAs, such as mRNA (2% by kinds and 0.4% by counts) and rRNA (3% by kinds and 0.4% by counts), indicating that the sequencing samples were not contaminated by degraded RNA and were therefore of high integrity. The analyses revealed that the majority of the non-redundant sequences, 20–24 nt in length, were the typical products processed from the enzyme Dicer (Fig 2A).

To characterize the shrimp miRNA homologues, the small RNA sequences were blasted against the miRbase 15.0 and shrimp EST database from GenBank with an E-value cutoff of 10 for the similarity. In total, 81 miRNAs were revealed (Fig 2B and Table S1, S2 and S3). The sequence analyses indicated that a total of 54 miRNAs were conserved in animals, which could be classified into 45 distinct families (Fig 2B and Table S1 and S2). The remaining miRNAs with no homologue in animals were blasted against the shrimp EST database allowing one mismatch between two sequences. After removal of those matched the Rfam database and mRNA sequences, the mapped EST sequences were used to predict the hairpin structures which were one of the key features to distinguish miRNAs from other endogenous small non-coding RNAs. According to this criterion, a total of 27 miRNAs with no homologue in animals were identified (Fig 2B and Table S3), which might be specific in shrimp or novel in animals. For the shrimp treated with the inhibitors or activators of apoptosis, phagocytosis or phenoloxidase, some of their miRNAs were different from each other (Fig 2B and Table S1), indicating the differences of shrimp miRNAs associated with the different innate immune responses.

### The miRNAs Involved in Apoptosis, Phagocytosis and pro-phenoloxidase System

**Figure 3:**
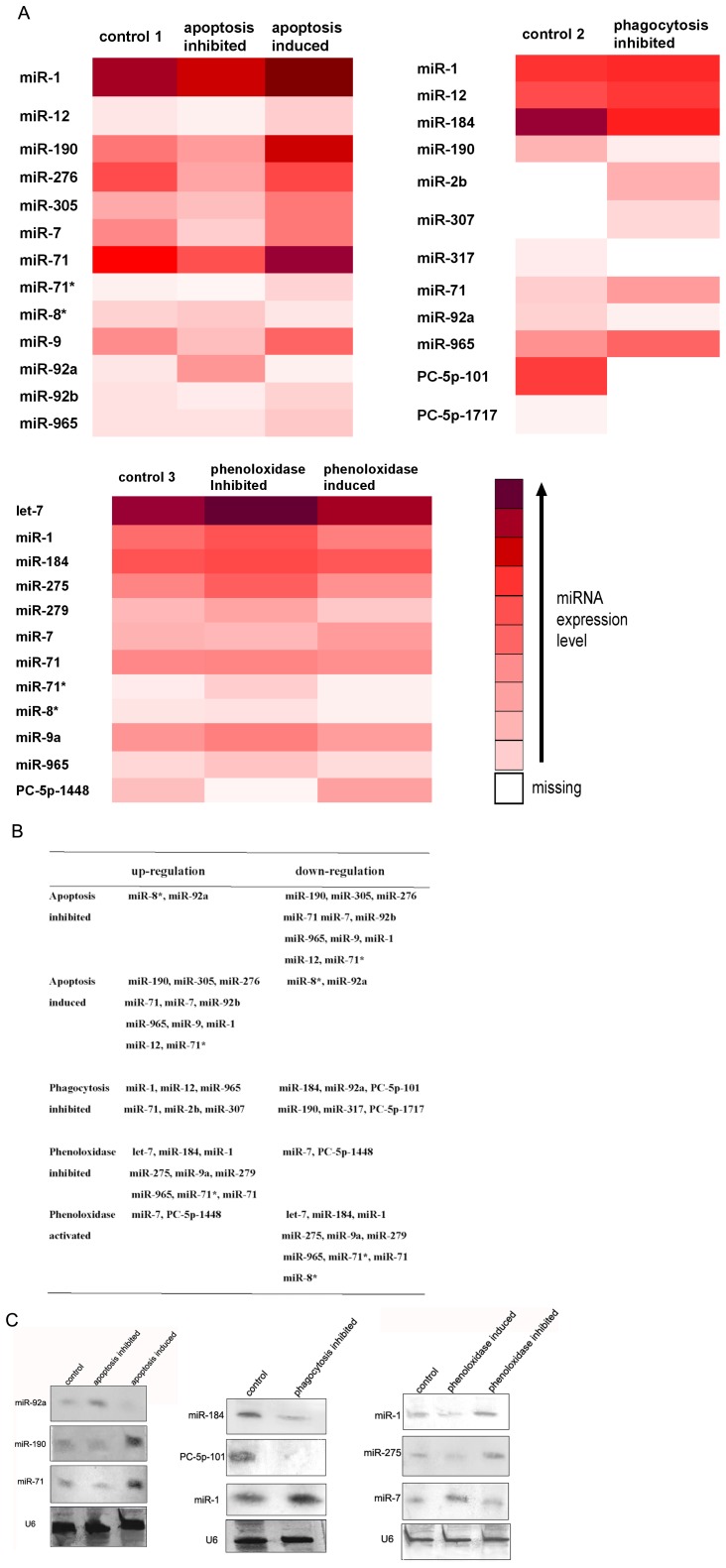
The shrimp miRNAs involved in apoptosis, phagocytosis and pro- phenoloxidase system. (**A**) The miRNA expression profiles in responses to the inhibition or activation of apoptosis, phagocytosis or phenoloxidase, respectively. The miRNAs of shrimp without immune inhibitor or activator were used as controls. Missing, not detected. (**B**) The miRNAs involved in apoptosis, phagocytosis or pro-phenoloxidase system. (**C**) Northern blots of miRNAs involved in apoptosis, phagocytosis or pro-phenoloxidase system. Total RNAs extracted from the hemolymph of shrimp treated with inhibitors or activators of apoptosis, phagocytosis or phenoloxidase were blotted with DIG-labeled oligodeoxynucleotide probes, respectively. The miRNAs of shrimp without immune inhibitor or activator were used as controls. The probes were shown at the left. The U6 was used as a loading control.

To identify the miRNAs involved in innate immune responses, the expression profiles of miRNAs of shrimp treated with inhibitors or activators of apoptosis, phagocytosis or phenoloxidase were compared, respectively. Based on the relative abundances of the miRNAs as sequenced, the results showed that the expression patterns of many miRNAs did not significantly change in response to the immune inhibitors or activators, while a total of 24 miRNAs were differentially expressed (Fig 3A , Table S4).

Among the 24 differentially expressed miRNAs, 13, 12 and 12 miRNAs might be involved in the regulation of apoptosis, phagocytosis and pro-phenoloxidase system, respectively (Fig 3A, Table S4). For apoptosis, it was found that, by comparison with the control, the miR-8* and miR-92a were up-regulated when the apoptosis was inhibited, while the two miRNAs were down-regulated when the apoptosis was induced (Fig 3A and 3B), indicating that the miR-8* and miR-92a might play crucial roles in the negative regulation of apoptosis. On the other hand, 11 miRNAs were down-regulated or up-regulated in responses to the inhibition or activation of apoptosis (Fig 3A and 3B), suggesting that miR-190, miR-305, miR-276, miR-71 miR-7, miR-92b, miR-965, miR-9, miR-1, miR-12 and miR-71* were required for the activation of apoptosis. For phagocytosis, 6 miRNAs were up-regulated and 6 miRNAs down-regulated when the phagocytotic activity was inhibited (Fig 3A and 3B). The data showed that miR-1, miR-12, miR-965, miR-71, miR-2b and miR-307 might be related to the negative regulation of phagocytosis and that miR-184, miR-92a, PC-5p-101, miR-190, miR-317 and PC-5p-1717 might positively regulate the phagocytic activity. For the pro-phenoloxidase system, when the hemocytic phenoloxidase activity was inhibited, 10 miRNAs (let-7, miR-184, miR-1, miR-275, miR-9a, miR-279, miR-965, miR-71*, miR-71 and miR-8*) were up-regulated, while they were down-regulated when the phenoloxidase activity was activated (Fig 3A and 3B), suggesting that these miRNAs played important roles in the negative regulation of pro-phenoloxidase system. It was revealed that the miR-7 and PC-5p-1448 were up-regulated or down-regulated when the phenoloxidase activity was activated or inhibited (Fig 3A and 3B), indicating that the two miRNAs might be essential for the activation of pro-phenoloxidase system.

To confirm the miRNAs involved in apoptosis, phagocytosis or pro-phenoloxidase system, 9 out of the 24 differentially expressed miRNAs were selected at random for Northern blots. The results showed that the expression patterns of miRNAs exhibited the similar profiles as those of miRNA sequencing analyses for apoptosis, phagocytosis or phenoloxidase, respectively (Fig 3C). The data presented that the 24 miRNAs might play important roles in the innate immunity including apoptosis, phagocytosis and pro-phenoloxidase system.

### The Pathways Mediated by the Innate Immunity-associated miRNAs

**Figure 4:**
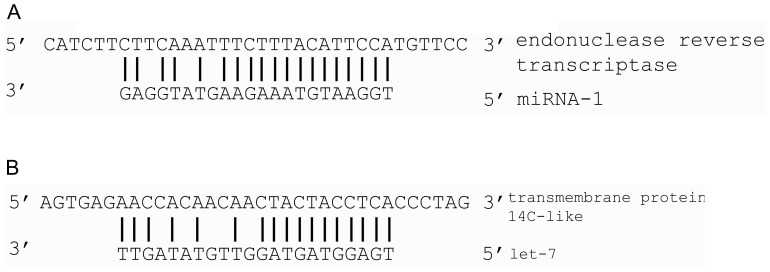
Target genes of miRNAs by degradome sequencing. (**A**) The target gene of miR-1. The arrow represented the splice site. (**B**) The target gene of lft-7. The arrow represented the splice site.

In an attempt to reveal the target genes of miRNAs, the degradome sequencing of shrimp was conducted. After multiplexed deep sequencing of degradome, a total of 26,654,540 sequences were obtained, of which 550,357 were unique reads. To distinguish the miRNA-directed cleavage from the cleavage events by other endonucleases, the reads that did not map to regions of the predicted miRNA basepairing were left out. In total, 9 miRNA-guided cleavages in shrimp hemocyte were identified. The results showed that 2 miRNAs (miR-1 and let-7) of the 24 innate immunity-associated miRNAs were included in the 9 miRNA-guided cleavages (Fig 4). The degradome sequencing analysis indicated that the target genes of miR-1 and let-7 were endonuclease-reverse transcriptase and transmembrane protein 14C-like, respectively (Fig 4, Table 1). The results showed that miR-1 might take effects in the regulation of phagocytosis, apoptosis and pro-phenoloxidase system by targeting the endonuclease-reverse transcriptase gene (Table 1).

**Table 1:** Pathways mediated by the innate immunity-associated miRNAs.

miRNA	Target genes^#^	Pathways
let-7	transmembrane protein 14C-like	Phenoloxidase
miR-1	endonuclease-reverse transcriptase	apoptosis, phagocytosis phenoloxidase
miR-12	signaling (initiator) caspase; histamine-gatedchloride channel subunit 1	apoptosis, phagocytosis
miR-184	alpha-2 macroglobulin; transferrin 2; transforming growth factor-beta receptorligand	phagocytosis, phenoloxidase
miR-190	heparan sulfate 3-O sulfotransferase-A;choline acetyltransferase	apoptosis, phagocytosis
miR-275	tyrosine-tRNA ligase; Rh7	Phenoloxidase
miR-276	Zn finger homeodomain 2; mannosyl-oligosaccharide glucosidase	Apoptosis
miR-279	nervous fingers 1; alpha-1,6-mannosyl-glycoproteinbeta-1,2-N-acetylgluco- saminyltransferase	phenoloxidase
miR-2b	high affinity inorganic phosphate	Phagocytosis
miR-305	transport and Golgi organization 13;grappa	Apoptosis
miR-307	general receptor for phosphoinositides 1;costa; microtubule binding/kinesin motor	Phagocytosis
miR-317	ethanolamine kinase; polychaetoid; guanylate cyclase; pyruvate dehydrogenase	phagocytosis
miR-7	region transcript m5; deltex; SH3-domain binding; Twin of m4	phenoloxidase, apoptosis
miR-71*	Null	Phenoloxidase
miR-71	Loss of InTestine; abnormal cell LINeage;GEX Interacting protein; Hunch Back Like	apoptosis, phagocytosis, phenoloxidase,
miR-8*	Null	Apoptosis
miR-9	nervous fingers 1; serpent;DNA binding	Apoptosis
miR-92a	cyclic-AMP response element bindingprotein A; Kinesin-73; Numb-associatedkinase	apoptosis, phagocytosis
miR-92b	cyclic-AMP response element bindingprotein A; Kinesin-73	apoptosis
miR-965	serotonin receptor 1B; string; ankyrin 2;target of rapamycin	apoptosis, phagocytosis, phenoloxidase,
miR-9a	Protein tyrosine phosphatase 69D;tubulinyl-tyrosine ligase	phenoloxidase
PC-5p-101	Null	Phagocytosis
PC-5p-1448	Null	Phenoloxidase
PC-5p-1717	Null	Phagocytosis

#, The target genes of miR-1 and let-7 were obtained by degradome sequencing and the targets of the remaining miRNAs were predicted.

Null, No target gene was predicted.

To get more information about the target genes of miRNAs, the TargetScan and miRanda algorithms were used to predict the miRNA targets. The results showed that the target genes of 19 innate immunity-associated miRNAs except for miR-1 and let-7 were available (Table 1). Based on the investigations in this study, the 24 innate immunity-associated miRNAs were involved in different immune pathways including phagocytosis, apoptosis and/or pro-phenoloxidase system, respectively (Table 1). The data revealed that the innate immunity-associated miRNAs might mediate the regulations of phagocytosis, apoptosis or pro-phenoloxidase system by targeting different genes.

## Discussion

The innate immune system of animals provides the immediate defense against the infection of pathogens in a non-specific manner, which is the first defense line found in vertebrates and invertebrates. Apoptosis, phagocytosis and pro-phenoloxidase system are considered to be the most important innate immune responses in the innate immunity, which are fine regulated by complicated systems mainly concerning to gene expression regulations. As well known, the miRNAs take crucial effects on the regulation of gene expression. In this context, the roles of miRNAs in the regulations of apoptosis, phagocytosis and pro-phenoloxidase system merit to be addressed. However, a comprehensive view about the regulation of innate immunity by miRNAs is not available at present. In this study, it was found that a total of 24 miRNAs might be involved in the innate immune responses including apoptosis, phagocytosis and pro-phenoloxidase system. Our study presented the first comprehensive view of the miRNAs associated with innate immunity. Nevertheless, our study provided only indications of the miRNA panorama related to innate immune responses of animals. Among the 24 miRNAs, the homology analysis showed that 21 miRNAs were conserved in animals. These miRNAs might play the similar roles in different animals. As revealed in this study, the miR-9 was up-regulated or down-regulated when the shrimp apoptosis was induced or inhibited, suggesting that the miR-9 participated in the apoptosis pathway and acted as an activator of apoptosis. It was reported that the miR-9 was highly expressed in most epithelial cells in the wing disc of *Drosophila* [22]. The miR-9 could prevent apoptosis during wing development by repressing *Drosophila* LIM-only. As documented, loss of one copy of *dLMO* rescued the apoptosis and wing margin defects in miR-9a mutants, suggesting that the miR-9 was involved in apoptosis and was associated with cell development [22]. In human ovarian tumor cells, the miR-9 was down-regulated and the over-expression of miR-9 suppressed their proliferation [23], [24], which might be in part due to the regulation of apoptosis by miR-9. The documented data suggested that the miRNAs conserved in animals shared the same or similar functions in different species of animals. Therefore the 24 innate immunity-associated miRNAs revealed in this study would be very helpful to reveal the molecular events in the regulations of innate immunity of animals. Our study revealed that two star miRNAs (miR-8* and miR-71*) might be associated with the regulation of innate immunity. The mature miRNA and its star miRNA which is complementary to the mature miRNA are generated from the same precursor miRNA. In general, the mature miRNA strand is liberated from the miRNA:miRNA* duplex and incorporated into the RNA-induced silencing complex (RISC). The star miRNA is supposed to be degraded. In some cases, however, it is reported that the star miRNA can also be loaded into RISC and take effects on gene expressions [25]. In this context, the miR-8* and miR-71* might play roles in the regulation of apoptosis or phenoloxidase.

It is well known that a miRNA functions by targeting its target genes. To get the targets of the innate immunity-associated miRNAs, a genome-wide degradome sequencing that captured RNAs bearing a 5′-phosphate termini was employed in our study. As evidenced, microRNAs could operate largely without the need for an intact Ago catalytic site. The Ago2-mediated cleavage, directly by miRNAs in plants and by siRNAs in animals, left a 5′-phosphorylated fragment to the cleavage site which could be sequenced by parallel analysis of RNA ends (PARE). Thus, the repertoire of microRNA-directed mRNA targets could be determined with degradome sequencing. The degradome sequencing has been widely used to validate the miRNA targets in plants. Recently, it is applied to identify the miRNA-derived cleavages in human and mouse [26], [27], [28]. In this study, the degradome sequencing was firstly employed to get the target genes of miRNAs in invertebrates. The results showed that the targets of miR-1 and let-7 from the 24 innate immunity-associated miRNAs were obtained, indicating that the degradome sequencing was an efficient strategy for the identification of target genes of miRNAs in invertebrates. Among the 24 innate immunity-associated miRNAs, only 2 miRNAs were assayed to get their targets by degradome sequencing. This might be caused due to the lack of shrimp genome sequence.

It is evidenced that the actin protein plays an essential role in the phagocytosis pathway. In phagocytosis, actin is reassembled to form pseudopodia, which could wrap particles on the cell surface and engulf them into cells. As reported, cytochalasin B can specially bind to the positive side of microfilaments, leading to the F-actin depolymerization and the block of polymerization of subunits [29]. Thus it can effectively reduce the phagocytic efficiency by inhibiting the assembly of actin dynamics. At present, however, there is no strategy to effectively enhance the activity of phagocytosis. Therefore the differentially expressed miRNAs in response to the activation of phagocytosis were not available in our study. This merited to be further investigated.

## Supporting Information

Table S1 **The shrimp miRNAs associated with innate immunity.**
(DOC)

Table S2 **The shrimp miRNAs conserved in animals.**
(DOC)

Table S3 **The shrimp miRNAs with no homologue in animals.**
(DOC)

Table S4 **The expression levels of miRNAs in responses to the inhibition or activation of apoptosis, phagocytosis or phenoloxidase.**
(DOC)
