# Supplementary material for: Signature miRNAs Involved in the Innate Immunity of Invertebrates
Source: PLoS One. 2012 Jun 19;7(6):e39015. doi: 10.1371/journal.pone.0039015 (PMC3378607; doi:10.1371/journal.pone.0039015)
Supplement: Table S1 — The shrimp miRNAs associated with innate immunity. (DOC) [file pone.0039015.s001.doc]

**Table S1. The shrimp miRNAs associated with innate immunity**

|  | miRNAs conserved in animals | miRNAs with no homologue in animals |  |
| --- | --- | --- | --- |
| control 1 | miR-iab,let-7,bantam,miR-9b,miR-9a,miR-998,miR-995,miR-993,miR-965,miR-92b,miR-92a,miR-92,miR-87,miR-8,miR-8*,miR-79,miR-7,miR-750,miR-745,miR-71,miR-71*,miR-34,miR33,miR-317,miR-315,miR-307,miR-306,miR-305,miR-2c,miR-2b,miR-2a,miR-283,miR-282,miR-28,1miR-279,miR-276,bmiR-276a,miR-276,miR-275,miR-263a,miR-252,miR-190,miR-184,miR-1miR-13a,miR-133,miR-12,miR-125,miR-124,miR-10a,miR-100,miR-1000,miR-10* | PC-3p-73,PC-5p-315,PC-5p-190 |  |
| apoptosis inhibited | miR-iab,let-7,bantam,miR-9b,miR-9a,miR-998,miR-995,miR-993,miR-965,miR-92b,miR-92a,miR-92,miR-87,miR-8,miR-8*,miR-79,miR-7,miR-750,miR-745,miR-71,miR-71*,miR-34,miR33,miR-317,miR-315,miR-307,miR-306,miR-305,miR-2c,miR-2b,miR-2a,miR-283,miR-282,miR-28,1miR-279,miR-276,bmiR-276a,miR-276,miR-275,miR-263a,miR-252,miR-190,miR-184,miR-1,miR-13a,miR-133,miR-12,miR-125,miR-124,miR-10a,miR-100,miR-1000,miR-10* | PC-5p-73,PC-5p-2058,PC-5p-1155,PC-5p-738,PC-5p-250,PC-5p-2685,PC-3p-2103 |  |
| apoptosis induced | miR-iab,let-7,bantam,miR-9b,miR-9a,miR-998,miR-995,miR-993,miR-965,miR-92b,miR-92a,miR-92,miR-87,miR-8,miR-8*,miR-79,miR-7,miR-750,miR-745,miR-71,miR-71*,miR-34,miR33,miR-317,miR-315,miR-307,miR-306,miR-305,miR-2c,miR-2b,miR-2a,miR-283,miR-282,miR-28,1miR-279,miR-276,bmiR-276a,miR-276,miR-275,miR-263a,miR-252,miR-190,miR-184,miR-1miR-13a,miR-133,miR-12,miR-125,miR-124,miR-10a,miR-100,miR-1000,miR-10* | PC-5p-250,PC-3p-247,PC-5p-73,PC-5p-783,PC-5p-1155,PC-5p-1629,PC-5p-1646,PC-5p-2103,PC-5p-2131,PC-5p-2249,PC-5p-2312,PC-5p-2353,PC-5p-2451 |  |
| control 2 | miR-iab,let-7,bantam,miR-9b,miR-9a,miR-998,miR-995,miR-993,miR-965,miR-92b,miR-92a,miR-92,miR-87,miR-8,miR-8*,miR-79,miR-7,miR-750,miR-745,miR-71,miR-71*,miR-34,miR33,miR-317,miR-315,miR-307,miR-306,miR-305,miR-2c,miR-2b,miR-2a,miR-283,miR-282,miR-28,1miR-279,miR-276,bmiR-276a,miR-276,miR-275,miR-263a,miR-252,miR-190,miR-184,miR-1miR-13a,miR-133,miR-12,miR-125,miR-124,miR-10a,miR-100,miR-1000,miR-10* | PC-5p-576,PC-5p-783,PC-5p-3339 |  |
| phagocytosis inhihited | miR-iab,let-7,bantam,miR-9b,miR-9a,miR-998,miR-995,miR-993,miR-965,miR-92b,miR-92a,miR-92,miR-87,miR-8,miR-8*,miR-79,miR-7,miR-750,miR-745,miR-71,miR-71*,miR-34,miR33,miR-317,miR-315,miR-307,miR-306,miR-305,miR-2c,miR-2b,miR-2a,miR-283,miR-282,miR-28,1miR-279,miR-276,bmiR-276a,miR-276,miR-275,miR-263a,miR-252,miR-190,miR-184,miR-1miR-13a,miR-133,miR-12,miR-125,miR-124,miR-10a,miR-100,miR-1000,miR-10* | PC-5p-6870 |  |
| control 3 | miR-iab,let-7,bantam,miR-9b,miR-9a,miR-998,miR-995,miR-993,miR-965,miR-92b,miR-92a,miR-92,miR-87,miR-8,miR-8*,miR-79,miR-7,miR-750,miR-745,miR-71,miR-71*,miR-34,miR33,miR-317,miR-315,miR-307,miR-306,miR-305,miR-2c,miR-2b,miR-2a,miR-283,miR-282,miR-28,1miR-279,miR-276,bmiR-276a,miR-276,miR-275,miR-263a,miR-252,miR-190,miR-184,miR-1miR-13a,miR-133,miR-12,miR-125,miR-124,miR-10a,miR-100,miR-1000,miR-10* | PC-3p-73,PC-5p-315,PC-5p-190 | |
| phenoloxidase inhibited | miR-iab,let-7,bantam,miR-9b,miR-9a,miR-998,miR-995,miR-993,miR-965,miR-92b,miR-92a,miR-92,miR-87,miR-8,miR-8*,miR-79,miR-7,miR-750,miR-745,miR-71,miR-71*,miR-34,miR33,miR-317,miR-315,miR-307,miR-306,miR-305,miR-2c,miR-2b,miR-2a,miR-283,miR-282,miR-28,1miR-279,miR-276,bmiR-276a,miR-276,miR-275,miR-263a,miR-252,miR-190,miR-184,miR-1miR-13a,miR-133,miR-12,miR-125,miR-124,miR-10a,miR-100,miR-1000,miR-10* | PC-5p-783,PC-5p-576,PC-5p-3339,PC-3p-1420,PC-5p-1833,PC-5p-2330,PC-3p-2353,PC-3p-2493 | |
| phenoloxidase activated | miR-iab,let-7,bantam,miR-9b,miR-9a,miR-998,miR-995,miR-993,miR-965,miR-92b,miR-92a,miR-92,miR-87,miR-8,miR-8*,miR-79,miR-7,miR-750,miR-745,miR-71,miR-71*,miR-34,miR33,miR-317,miR-315,miR-307,miR-306,miR-305,miR-2c,miR-2b,miR-2a,miR-283,miR-282,miR-28,1miR-279,miR-276,bmiR-276a,miR-276,miR-275,miR-263a,miR-252,miR-190,miR-184,miR-1miR-13a,miR-133,miR-12,miR-125,miR-124,miR-10a,miR-100,miR-1000,miR-10* | PC-5p-576,PC-5p-783,PC-5p-1798,PC-5p-3337,PC-5p-3339,PC-3p-3431,PC-3p-4269,PC-3p-1420 | |

Controls 1, 2 and 3: shrimp without inhibitors or activators of apoptosis, phagocytosis or phenoloxidase
