# Supplementary material for: Signature miRNAs Involved in the Innate Immunity of Invertebrates
Source: PLoS One. 2012 Jun 19;7(6):e39015. doi: 10.1371/journal.pone.0039015 (PMC3378607; doi:10.1371/journal.pone.0039015)
Supplement: Table S2 — The shrimp miRNAs conserved in animals. (DOC) [file pone.0039015.s002.doc]

**Table S2. The shrimp miRNAs conserved in animals**

| Name | Sequence (5’-3’) | Length (nt) |
| --- | --- | --- |
| miR-iab | ACGTATACTGAATGTATCCTGA | 22 |
| let-7 | TGAGGTAGTCGGTTGTATAGT | 21 |
| Bantam | TGAGATCATTGTGAAAGCTGATT | 23 |
| miR-9b | TCTTTGGTGATTTAGCTGTATG | 22 |
| miR-9a | TCTTTGGTGATCTAGCTGTATGA | 23 |
| miR-998 | TAGCACCATGAGATTCAGCTA | 21 |
| miR-995 | TAGCACCACATGATTCAGC | 19 |
| miR-993 | ACCCTGTAGACACCGGGCTTTTG | 23 |
| miR-965 | TAAGCGTATGGCTTTTCCCCT | 21 |
| miR-92b | AATTGCACTAGTCCCGGCCTG | 21 |
| miR-92a | TATTGCACTTGTCCCGGCCTGT | 22 |
| miR-92 | AATTGCACTCGTCCCGGCCTGC | 22 |
| miR-87 | GTGAGCAAAGTTTCAGGTGTGT | 22 |
| miR-8 | TAATACTGTCAGGTAAAGATGTA | 23 |
| miR-8* | CATCTTACCGGACAGCATTAGA | 22 |
| miR-79 | ATAAAGCTAGATTACCAAAGCA | 22 |
| miR-7 | TGAGGTAGTAGGTTGTATAGTT | 22 |
| miR-750 | CCAGATCTAACTCTTCCAGCTCA | 23 |
| miR-745 | GAGCTGCCCAATGAAGGGCT | 20 |
| miR-71 | TGAAAGACATGGGTAGTGAGAT | 22 |
| miR-71* | TCTCACTACCTTGTCTTTCACG | 22 |
| miR-34 | TGGCAGTGTGGTTAGCTGGTT | 21 |
| miR33 | ATGCATTGTAGTTGCATTGCA | 21 |
| miR-317 | TGAACACAGCTGGTGGTATCTCA | 23 |
| miR-315 | TTTTGATTGTTGCTCAGAAGGC | 22 |
| miR-307 | TCACAACCTCCTTGAGTGAGTGA | 23 |
| miR-306 | TCAGGTACTATGTGACTCTG | 20 |
| miR-305 | ATTGTACTTCATCAGGTGCTCGG | 23 |
| miR-2c | TATCACAGCCAGCTTTGATG | 20 |
| miR-2b | TATCACAGCCACCTTTGATGAGCT | 24 |
| miR-2a | TATCACAGCCAGCTTTGATGAGCG | 24 |
| miR-283 | AAATATCAGCAGGTAATTT | 19 |
| miR-282 | TAGCCTCTCCTTGGCTTTGTCT | 22 |
| miR-281 | AAGAGAGCTATCCGTCGACAGT | 22 |
| miR-279 | TGACTAGATCCACACTCATCCA | 22 |
| miR-276b | TAGGAACTTTATACCGTGCTCT | 22 |
| miR-276a | TAGGAACTTCATACCGTGCTCTT | 23 |
| miR-276 | TAGGAACTTCATACCGTGCTCT | 22 |
| miR-275 | TCAGGTACCTGATGTAGCGCGCG | 23 |
| miR-263a | AATGGCACTGGAAGAATTCACGG | 23 |
| miR-252 | CTAAGTACTAGTGCCGCAGGAG | 22 |
| miR-190 | AGATATGTTTGATATTCTTGGTTG | 24 |
| miR-184 | TGGACGGAGAACTGATAAGGGC | 22 |
| miR-1 | TGGAATGTAAAGAAGTATGGAG | 22 |
| miR-13a | TATCACAGCCACCTTTGATGAGCT | 24 |
| miR-133 | TTGGTCCCCTTCAACCAGCTGT | 22 |
| miR-12 | TGAGTATTACATCAGGTACTGGT | 23 |
| miR-125 | TCCCTGAGACCCTAACTTGTGA | 22 |
| miR-124 | TAAGGCACGCGGTGAATGCCA | 21 |
| miR-10a | TACCCTGTAGATCCGAATTTGT | 22 |
| miR-100 | AACCCGTAGATCCGAACTTGTG | 22 |
| miR-1000 | ATATTGTCCCGTCACAGCAGTA | 22 |
| miR-10* | AAATTCGGTTCTAGAGAGGTTT | 22 |
