# Supplementary material for: Signature miRNAs Involved in the Innate Immunity of Invertebrates
Source: PLoS One. 2012 Jun 19;7(6):e39015. doi: 10.1371/journal.pone.0039015 (PMC3378607; doi:10.1371/journal.pone.0039015)
Supplement: Table S3 — The shrimp miRNAs with no homologue in animals. (DOC) [file pone.0039015.s003.doc]

**Table S3.** **The shrimp miRNAs with no homologue in animals**

| Name | Sequence (5’-3’) | Length (nt) |
| --- | --- | --- |
| PC-3p-4269 | TGCTTTAAGAAGACTTATTACTT | 23 |
| PC-5p-2131 | TCCTTAGAGTCGGGTTCTATC | 21 |
| PC-5p-73 | GTTCGCCGTAGAGCACCGCGCAA | 23 |
| PC-5p-2058 | GTGCGGCGCAACGGATCGGCAA | 22 |
| PC-5p-2451 | GGGGGTCGTTGGCCGTAACCT | 21 |
| PC-5p-315 | GGGGGCAGTCCGTACCTGACT | 21 |
| PC-5p-1155 | GGGAGTGCGGAACGGGCCTC | 20 |
| PC-5p-1833 | GGGACTCGCCATGACGAAC | 19 |
| PC-5p-2249 | GGCGCAACGGATCGGCGATT | 20 |
| PC-5p-783 | GGCACCGGACTGGCGCCCTT | 20 |
| PC-5p-2330 | GCGGGTAACCTCGTGAAAGCTGT | 23 |
| PC-5p-2353 | GCAGGACGGTGGCCATGGAAGTTG | 24 |
| PC-5p-1717 | GATAGGTGAGAGTCTGGTACATG | 23 |
| PC-5p-1646 | GATAATGATGATGGTGCTGATG | 22 |
| PC-5p-1798 | CTTGACTCTAGTTTGGCTTTGTA | 22 |
| PC-5p-2312 | CGGGTCGACGGAAAGGTGTCCAA | 23 |
| PC-5p-1448 | CGGCGGTAGCCCGGGCAATAAG | 22 |
| PC-3p-1420 | CGATAGATCAATGTAGGTAAGG | 22 |
| PC-5p-2103 | CCCCTTGTGGCCGGCACGCAT | 21 |
| PC-5p-6870 | CACCAGGCCCGGACACCACCT | 21 |
| PC-3p-2353 | ATTAGAGTGCTCAGAGCAGGCT | 22 |
| PC-5p-3337 | ATGGTCTAGTGAGGGCACCG | 20 |
| PC-5p-1629 | ATGGAAGTTGGAATCCTCAT | 20 |
| PC-5p-101 | ATACAAACATGACTTCTATCTA | 22 |
| PC-5p-3339 | AGGGGGAAACCGCGCTGAGCGTTA | 24 |
| PC-3p-2493 | AGGGCAAATGCTGGCTGAC | 19 |
| PC-3p-247 | ACGGTCGGATGGTTTTGTCCG | 21 |
