# Supplementary material for: Signature miRNAs Involved in the Innate Immunity of Invertebrates
Source: PLoS One. 2012 Jun 19;7(6):e39015. doi: 10.1371/journal.pone.0039015 (PMC3378607; doi:10.1371/journal.pone.0039015)
Supplement: Table S4 — The expression levels of miRNAs in responses to the inhibition or activation of apoptosis, phagocytosis or phenoloxidase. (DOC) [file pone.0039015.s004.doc]

**Table S4. The expression levels of miRNAs in responses to the inhibition or activation of apoptosis, phagocytosis or phenoloxidase.**

|  | control 1 | apoptosis inhibited | apoptosis induced |
| --- | --- | --- | --- |
| miR-1 | 1.00 | 0.82 | 1.67 |
| miR-12 | 1.00 | 0.76 | 2.69 |
| miR-190 | 1.00 | 0.58 | 4.80 |
| miR-276 | 1.00 | 0.34 | 1.34 |
| miR-305 | 1.00 | 0.54 | 2.19 |
| miR-7 | 1.00 | 0.27 | 1.33 |
| miR-71 | 1.00 | 0.58 | 2.63 |
| miR-71* | 1.00 | 0.65 | 1.68 |
| miR-8* | 1.00 | 1.21 | 0.41 |
| miR-9 | 1.00 | 0.37 | 1.56 |
| miR-92a | 1.00 | 5.91 | 0.04 |
| miR-92b | 1.00 | 0.31 | 1.81 |
| miR-965 | 1.00 | 0.80 | 1.78 |

|  | control 2 | phagocytosis inhibited |
| --- | --- | --- |
| miR-1 | 1.00 | 1.46 |
| miR-12 | 1.00 | 1.55 |
| miR-184 | 1.00 | 0.59 |
| miR-190 | 1.00 | 0.43 |
| miR-2b | missing |  |
| miR-307 | missing |  |
| miR-317 | 1.00 | 0.92 |
| miR-71 | 1.00 | 1.99 |
| miR-92a | 1.00 | 0.21 |
| miR-965 | 1.00 | 1.94 |
| PC-5p-101 | 1.00 | missing |
| PC-5p-1717 | 1.00 | missing |

|  | control 3 | phenoloxidase Inhibited | phenoloxidase induced |
| --- | --- | --- | --- |
| let-7 | 1.00 | 1.58 | 0.76 |
| miR-1 | 1.00 | 3.64 | 0.62 |
| miR-184 | 1.00 | 1.54 | 0.92 |
| miR-275 | 1.00 | 5.51 | 0.86 |
| miR-279 | 1.00 | 1.50 | 0.63 |
| miR-7 | 1.00 | 0.74 | 2.06 |
| miR-71 | 1.00 | 1.52 | 0.81 |
| miR-71* | 1.00 | 2.81 | 0.86 |
| miR-8* | 1.00 | 1.35 | 0.92 |
| miR-9a | 1.00 | 1.93 | 0.74 |
| miR-965 | 1.00 | 2.39 | 0.98 |
| PC-5p-1448 | 1.00 | 0.06 | 1.67 |

Note: The miRNAs of shrimp without immune inhibitor or activator were used as controls. missing, not detected.
